# VPS35-deficiency results in an impaired AMPA receptor trafficking and decreased dendritic spine maturation

**DOI:** 10.1186/s13041-015-0156-4

**Published:** 2015-10-31

**Authors:** Yun Tian, Fu-Lei Tang, XiangDong Sun, Lei Wen, Lin Mei, Bei-Sha Tang, Wen-Cheng Xiong

**Affiliations:** Department of Geriatrics and Department of Neurology, Xiangya Hospital, Central South University, ChangSha, 410008 China; Department of Neuroscience & Regenerative Medicine and Department of Neurology, Medical College of Georgia, Augusta, GA 30912 USA; Charlie Norwood VA Medical Center, Augusta, GA 30912 USA; Department of Traditional Chinese Medicine, Xia-Men University, Xia-Men, China

**Keywords:** VPS35, Retromer, AMPA receptor, Dendritic spine, Glutamatergic transmission

## Abstract

**Background:**

Vacuolar protein sorting 35 (VPS35), a key component of retromer, plays an important role in endosome-to-Golgi retrieval of membrane proteins. Dysfunction of VPS35/retromer is a risk factor for neurodegenerative disorders, including AD (Alzheimer’s disease) and PD (Parkinson’s disease). However, exactly how VPS35-deficiency contributes to AD or PD pathogenesis remains poorly understood.

**Results:**

We found that VPS35-deficiency impaired dendritic spine maturation and decreased glutamatergic transmission. AMPA receptors, GluA1 and GluA2, are significantly reduced in purified synaptosomal and PSD fractions from VPS35-deficient brain. The surface levels of AMPA receptors are also decreased in VPS35-deficient neurons. Additionally, VPS35 interacted with AMPA-type receptors, GluA1 and GluA2. Overexpression of GluA2, but not GluA1, could partially restore the spine maturation deficit in VPS35-deficient neurons.

**Conclusions:**

These results provide evidence for VPS35’s function in promoting spine maturation, which is likely through increasing AMPA receptor targeting to the postsynaptic membrane. Perturbation of such a VPS35/retromer function may contribute to the impaired glutamatergic transmission and pathogenesis of neurodegenerative disorders, such as AD and PD.

**Electronic supplementary material:**

The online version of this article (doi:10.1186/s13041-015-0156-4) contains supplementary material, which is available to authorized users.

## Background

Synapse formation, maintenance, and elimination are critical for normal neurotransmission. In the early stage of neurodegenerative diseases, such as Alzheimer’s disease (AD) and Parkinson’s disease (PD), synaptic neurotransmission is often defective. This synaptic deficit appears to be an earlier deficit than neuron-loss [1–3]. In AD patients, cognitive decline is more likely to be correlated with the synaptic deficit rather than neuronal-loss [2, 4]. Therefore, it is of considerably interest to investigate how synapses are altered in the course of neurodegenerative disorders.

Vacuolar protein sorting-35 (VPS35) is an important component of the cargo-recognition retromer complex that contains VPS35, VPS29 and VPS26 [5–7]. The retromer complex is in charge of sorting cargos into retrieval pathway from endosome to Golgi apparatus [5–7]. Recently, several lines of evidence implicate this cargo-recognition complex together with SNX27 and WASH complex in endosome-to cell surface recycling of membrane receptors [8, 9]. Retromer is also found to mediate fast, local delivery of β2-adrenergic receptors (β2ARs) from endosomes to the dendritic plasma membrane [10].

VPS35/retromer dysfunction appears to be a risk factor for neurodegenerative disorders, including AD and PD. [11–16] In late-onset AD patients, VPS35 level is lower in hippocampal region [14]. Hemizygous deletion of VPS35 enhances AD-like neuropathology in Tg2576 mouse model of AD [17]. VPS35 (also called PARK17) mutations are identified in autosomal dominant PD patients [11, 12]. VPS35 level is also decreased in substantia nigra of PD patients [18]. Recently, using VPS35 deficient mouse models, including VPS35 heterozygotes and selective deletion of VPS35 in dopamine neurons, we have demonstrated that loss of VPS35 function in dopamine neurons results in PD-relevant neuropathology [15, 16]. While these observations support the view for VPS35-deficiency as a risk factor for AD and PD, the underlying molecular pathological mechanisms appear to be a complex. Intriguingly, impaired postsynaptic glutamatergic neurotransmission has been detected in young adult VPS35-deficient hippocampus [17], and transient suppression of VPS35 expression in developing hippocampal neurons results in abnormal dendritic spines [19]. These observations suggest that VPS35 may play a role in spine formation and function, which may underlie VPS35-prevention of AD pathogenesis.

In this paper, we further investigated VPS35’s function in neuronal spine formation and maturation in culture and in vivo. We provided morphological and electrophysiological evidence for VPS35-deficiency to contribute to the spine deficit. In addition, VPS35 interacted with AMPA receptors (AMPARs), and VPS35-deficiency impaired AMPAR, particularly GluA1, surface targeting. Furthermore, expression of GluA2, but not GluA1, partially restored the spine deficit due to VPS35-deficiency. Taken together, our results suggest that VPS35 plays a crucial role in regulating AMPA receptor trafficking, which may underlie its function in promoting spine maturation and glutamatergic neurotransmission, revealing a molecular mechanisms by which VPS35/retromer in hippocampal neurons prevents AD-neuro-pathogenesis.

## Results

### Spine maturation deficit in VPS35-deficient brain

**Fig. 1:**
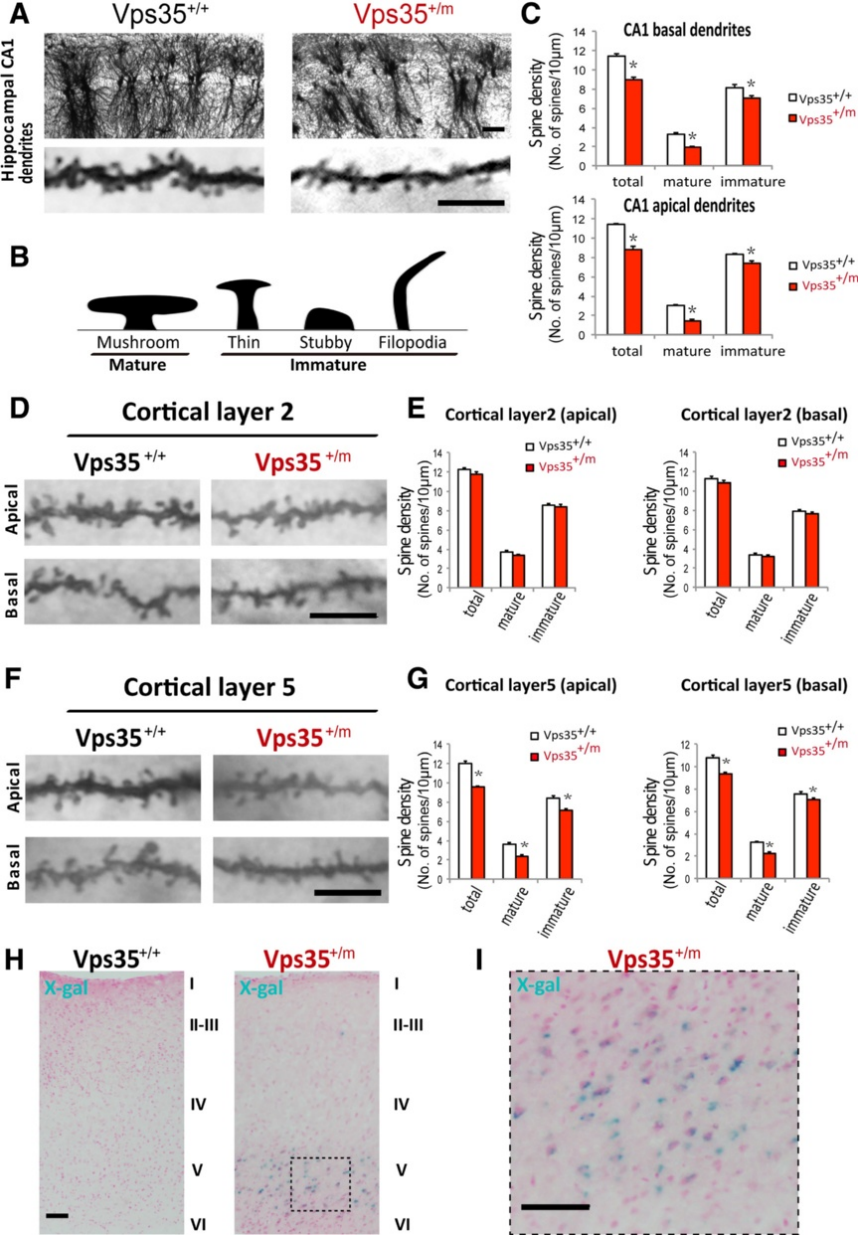
Reduced mature spines in VPS35^+/m^ hippocampus and cortex. **a** Reduced mature spines in both apical and basal dendrites of CA1 region from VPS35^+/m^ mice at age of 4-months old by Golgi staining analysis. Top, representative lower power images. Bottom, higher power images of apical dendrites from CA neurons. Scale bars, 5 μm. **b** Quantification of spine density and spine morphology in CA1 basal and apical dendrites viewed by Golgi staining analysis. Data shown were mean ± SEM; *n* = 20 neurons; *p < 0.05. **c** Illustration of 4 different types of spines (mushroom, thin, stubby, and filopodia) based on their morphology. The mushroom spines were defined as mature spines, and the other three types of spines were defined as immature spines. **d**, **e** No difference in spine density and morphology in cortical layer 2 neurons between WT and VPS35^+/m^ mice at age of 4-months old. Scale bars, 5 μm. D, representative images of Golgi staining analysis; and E, quantification analysis. Data were shown as mean ± SEM; *n* = 20 neurons; *p < 0.05. **f**, **g**Reduced mature spines in both apical and basal dendrites of cortical layer 5 from 4 months old VPS35^+/m^ mice. F, representative images. Scale bars, 5 μm. G, quantification analysis. Data were shown as mean ± SEM; *n* = 20 neurons; *p < 0.05. **h** Detection of β-Gal activity (blue color) in VPS35^+/m^ cortical layer 5 at age of 3-months old. Layers I-VI were indicated. Left, Representative X-gal staining images. Right, Amplified images of cortical layer 5 from VPS35^+/m^ brain. Scale bars, 100 μm

By taking advantage of VPS35 heterozygous mice (VPS35^+/m^) that have the lacZ gene knocked-in into VPS35 gene, resulting in a 50 % reduction of VPS35 proteins [17, 20], we preformed Golgi staining analysis of neuronal dendritic spines in VPS35^+/+^ (WT controls) and VPS35^+/m^ mice (4 months old). Golgi staining analysis showed that the spine densities of both apical and basal dendrites of CA1 pyramidal neurons were reduced in VPS35^+/m^ hippocampus, compared with that of WT controls (Figs. 1a, b). Further examining mature and immature spines (revealed by spine morphology as illustrated in Fig. 1c) demonstrated an obvious reduction of mature spines in VPS35-deficient CA1 hippocampal neurons (Fig. 1a, b). No difference was observed in spine density or morphology in cortical layer 2 pyramidal neurons between VPS35^+/+^ and ^+/m^ brain (Fig. 1d, e). In contrast, the spine density reduction was detected in cortical layer 5 pyramidal neurons of VPS35^+/m^ brain (Fig. 1f, g). Based on β-Gal reporter analysis of VPS35^+/m^ brain, VPS35 was expressed in large in the cortical layer 5 and hippocampal CA1-3 pyramidal neurons (Fig. 1h, i) [17]. Thus, the region-selective spine deficit appeared to be correlated well with VPS35’s expression pattern.

### Spine maturation deficit in VPS35-deficient neurons

**Fig. 2:**
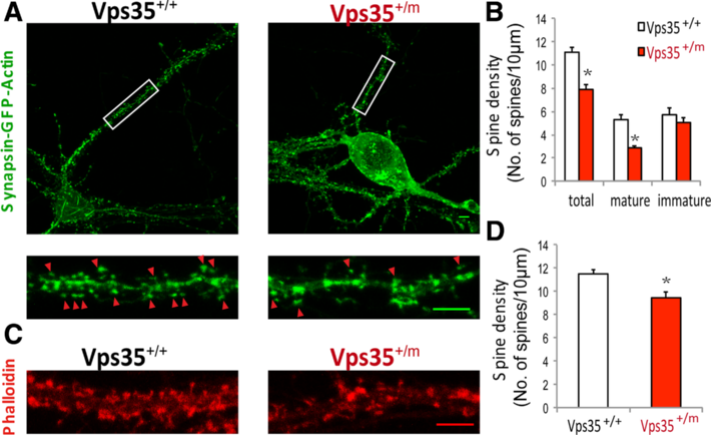
Reduced mature spines in VPS35^+/m^ neurons. **a** Reduced spine density and altered spine morphology in Synapsin-GFP-Actin transfected primary hippocampal neurons from VPS35^+/m^ mice compared with VPS35^+/+^ controls. Primary hippocampal neurons transfected with Synapsin-GFP-Actin at DIV7 were fixed at DIV15. Top, representative images. Bottom, amplified images. Scale bars, 5 μm. **b** Quantification of spine density and spine morphology in A. Data were shown as mean ± SEM; *n* = 30 neurons from 3 independent experiments; *p < 0.05. **c** Reduced spine density in primary VPS35^+/m^ hippocampal neurons viewed by phalloidin staining analysis. Primary hippocampal neurons were fixed at DIV18 and stained with phalloidin. Scale bars, 5 μm. **d** Quantification of spine density in C. Data were presented as mean ± SEM; *n* = 30 neurons from 3 independent experiments; *p < 0.05

We next addressed if VPS35 plays a cell autonomous role in promoting spine maturation in cultured neurons. The spine density and morphology were examined in primary cultured VPS35^+/+^ and VPS35^+/m^ hippocampal neurons. The reduction of VPS35 level in VPS35^+/m^ hippocampal neurons was confirmed by immunostaining analysis with VPS35 antibody (Additional file 1: Figure S1A). The spine morphology and density were revealed by the exogenous Synapsin promoter driven GFP-β-actin (Synapsin-GFP-β-actin) in VPS35^+/+^ and VPS35^+/m^ hippocampal neurons (transfected at DIV 10, and imaged at DIV 15). VPS35^+/m^ hippocampal neurons displayed reduced spine numbers per 10 μm, compared with that of WT control neurons (Fig. 2a, b). In particular, the mature spines such as mushroom spines were largely decreased (Fig. 2c). However, the number of filopodia-like spines, which are usually considered as immature spines, was increased in VPS35-deficient neurons (Fig. 2c). A similar spine deficit was also detected in VPS35^+/m^ hippocampal neurons at DIV 18 by phalloidin staining, which labels F-actin (Figs. 2d, e). Moreover, VPS35^+/m^ cortical neurons showed the similar spine defect (Additional file 1: Figure S1B). These results provide additional support for the necessity of VPS35 in neuronal spine maturation, supporting the view for a cell autonomous role of VPS35 in promoting spine maturation.

### Impaired glutamatergic transmission and decreased excitatory synapses in VPS35-deficient CA1 hippocampus

**Fig. 3:**
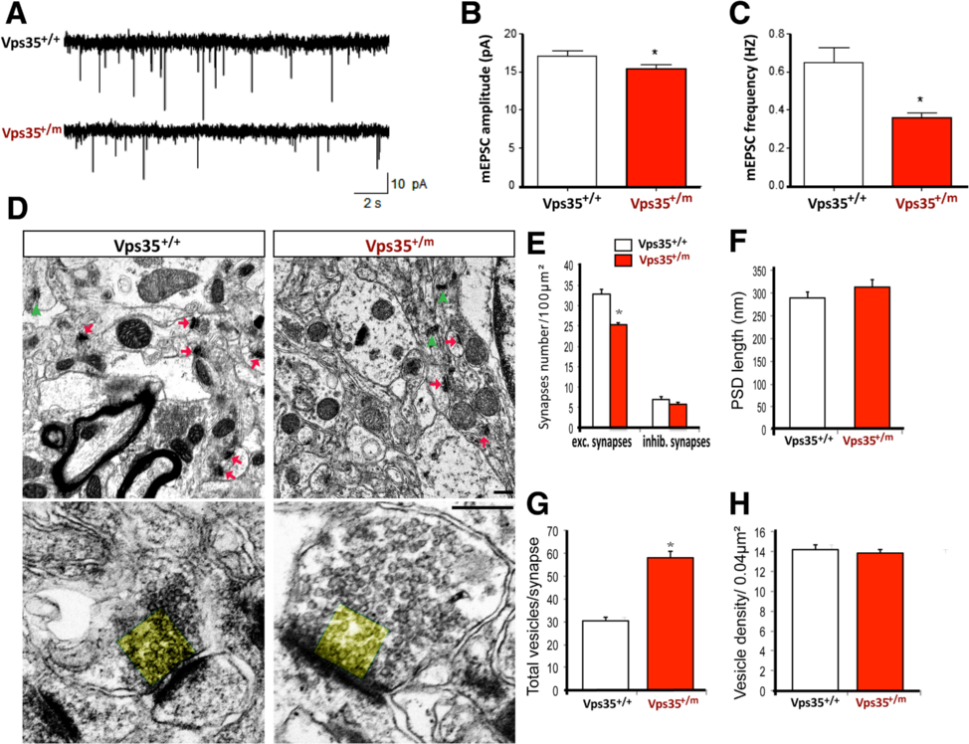
Impaired glutamatergic transmission, reduced excitatory synapses, and increased vesicles per synapse in hippocampus of VPS35^+/m^ mice. **a** Representative traces of mEPSCs in CA1 pyramidal neurons from VPS35^+/+^ and VPS35^+/m^ mice. Scale bar, 2 s, 10 pA. **b** Histograms of mEPSC amplitude. *n* = 15 neurons from 3 mice for each genotype. Student’s *t* test, *p < 0.05. **c** Histograms of mEPSC frequency. *n* = 15 neurons from 3 mice for each genotype. Student’s *t* test, *p < 0.05. **d** Decreased number of excitatory synapses and increased number of vesicles per synapse in 4-months old VPS35^+/m^ hippocampus. Top, representative transmission electron microscopic (TEM) images. Red arrows indicate excitatory synapse. Green arrows indicate inhibitory synapse. Scale bar, 200 nm. Bottom, Close up view of one excitatory synapses. **e**-**h** Quantification analyses of number of synapses (**e**), PSD length (**f**), number of vesicles per synapse(G), and vesicle density in 200 nm*200 nm square shown in yellow color near active zone in D (**h**). Data were shown as mean ± SEM; *n* = 30 images from 3 mice per genotype; *p < 0.05

The reduced excitatory spines in VPS35-deficient brain may result in an impaired glutamatergic transmission. To test this view, we recorded in whole-cell configuration the miniature excitatory postsynaptic currents (mEPSCs) from CA1 pyramidal neurons of 2-month-old hippocampus (Fig. 3a). A significant reduction of the frequency of mEPSCs in the mutant CA1 neurons was detected (Fig. 3a and c), in agreement with results from Golgi staining analysis that showed a reduced spine density in VPS35^+/m^ hippocampus (Fig. 1). In addition, the amplitude of mEPSCs was also reduced in the CA1 pyramidal neurons from VPS35^+/m^ brain, as compared with the WT controls (Fig. 3a, b). Note that the frequency of mEPSC was also lower in the mutant CA1 than that of WT controls (Fig. 3c), implicating a reduced presynaptic vesicular glutamate release, or a decreased numbers of synapse. Given the fact that paired-pulse facilitation (PPF) ratio was unchanged in the VPS35^+/m^ hippocampus [17], which indicates normal probability of presynaptic vesical release, the reduced frequency may be due to a reduction in the numbers of excitatory synapses of mutant hippocampal neurons.

We thus further analyzed the number of synapses in the hippocampus by transmission electron microscopy (EM). The excitatory synapse was indeed decreased in VPS35^+/m^ hippocampus, compared with that of WT controls (Fig. 3d, e). No significant change in the inhibitory synapses was detected in the mutant hippocampus (Fig. 3d, e). The PSD (post-synaptic density) length and thickness appeared also normal without obvious difference (Fig. 3f). Interestingly, an increase in the number of vesicles per synapse in the mutant hippocampus was observed (Fig. 3g). As no difference is observed in the ratio of PPF between VPS35^+/+^ and VPS35^+/m^ CA1 pyramidal neurons [17], these increased numbers of vesicles may be non-releasable/functional vesicles. This view was further supported by the observation that the vesicle numbers near the active zone in a 200 nm*200 nm square (indicated in yellow in Fig. 3d) in the mutant hippocampus were comparable to that of WT controls (Fig. 3d, h). Taken together, these results support the view for VPS35 expression in hippocampal pyramidal neurons to be critical for the excitatory spine maturation and glutamatergic neurotransmission.

### Reduced AMPA receptors and decreased AMPA receptor cell surface distribution in VPS35-deficient brain and neurons

**Fig. 4:**
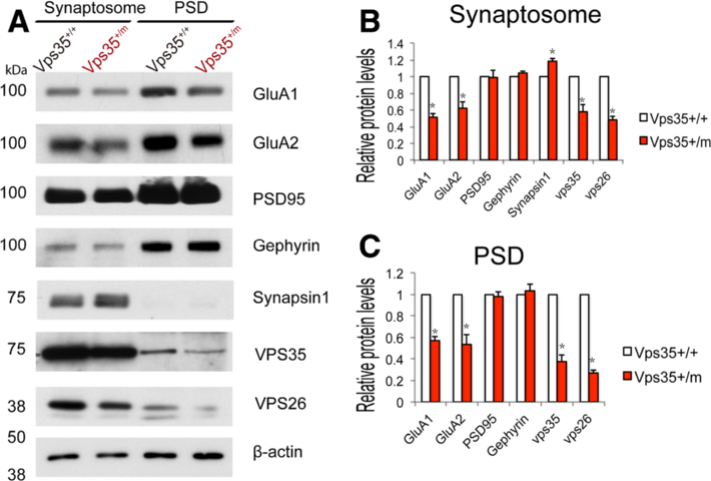
Reduced synapse associated GluA1 and GluA2 proteins in VPS35-deficient brain. **a** The glutamate receptor subunits GluA1 and GluA2’s levels were decreased in both synaptosomal and PSD fractions from 3-months old VPS35^+/m^ mouse brains. **b**, **c** Quantification of relative synapse associated protein levels in synaptosomal fraction (**b**) and PSD fraction (**c**). Data were presented as mean ± SEM; *n* = 3; *p < 0.05

To understand how VPS35 regulates glutamatergic neurotransmission and excitatory synapse/spine maturation, we examined protein levels of synapse-associated proteins in purified synaptosomal and PSD fractions from VPS35^+/+^ and VPS35^+/m^ cortex. Interestingly, GluA1 and GluA2 levels were reduced in both synaptosomal and PSD fractions of VPS35^+/m^ cortex, compared with WT controls, by Western blot analysis (Fig. 4a-c). A slight decrease in PSD95, the postsynaptic marker of excitatory synapse, was also detected (Fig. 4). No change in gephyrin level, a postsynaptic marker of inhibitory synapse, was observed (Fig. 4). In contrast, the synapsin-1, a presynaptic marker, was increased in synaptosomal fractions of VPS35^+/m^ cortex, compared with WT controls (Fig. 4), in line with the increased numbers of synaptic vesicles by EM studies.

**Fig. 5:**
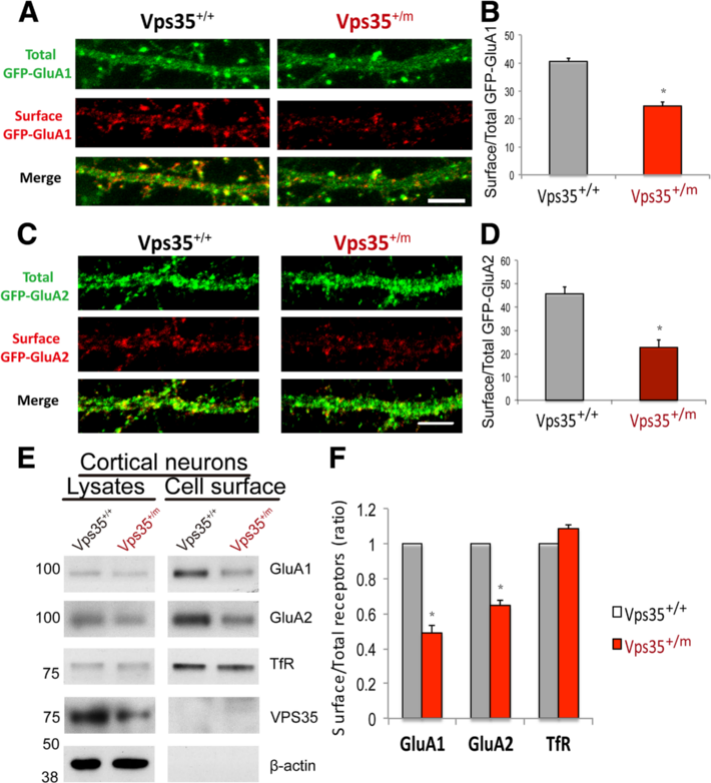
Decreased cell surface levels of GluA1 and GluA2 receptors in VPS35-deficient neurons. **a**, **b** Reduced surface GFP-GluA1 in VPS35^+/m^ hippocampal neurons. Both VPS35^+/+^ and VPS35^+/m^ hippocampal neurons were transfected with GFP-GluA1 at DIV7. The surface GFP-GluA1 was labeled by GFP antibody (monoclonal) under non-permeabilizing conditions (no Triton X-100) at DIV 15, then the neurons were fixed. The total GFP-GluA1 were labeled by GFP antibody (polyclonal) after permeablized by Triton X-100. A, representative imunnostaining images. Scale bars, 5 μm. B, quantification of surface to total GFP-GluA1 in A. Data were shown as mean ± SEM; *n* = 30 neurons from 3 independent experiments; *p < 0.05. **c**, **d** Reduced surface GFP-GluA2 in VPS35^+/m^ hippocampal neurons. As GFP-GluA1, GFP-GluA2 was transfected into VPS35^+/+^ and VPS35^+/m^ hippocampal neurons at DIV7. The surface and total GFP-GluA2 were labeled by GFP antibody as described above. C, representative imunnostaining images. Scale bars, 5 μm. D, quantification of surface to total GFP-GluA2 in C. Data were shown as mean ± SEM; *n* = 30 neurons from 3 independent experiments; *p < 0.05. **e**, **f** Reduced cell surface level of GluA1 and GluA2 in VPS35^+/m^ cortical neurons by biotinylation assay. E, representative blots; F, quantification of surface over total receptors (ratio) in E. Data were presented as mean ± SEM; *n* = 3; *p < 0.05

Both GluA1 and GluA2 are AMPARs, which are largely distributed in the postsynaptic membrane of excitatory synapse [21–24]. We thus further examined AMPARs’ distribution in cultured neurons from WT and VPS35^+/m^ brain. The exogenous GFP-GluA1 and GFP-GluA2 were used to view GluA1 and GluA2 distribution in WT and VPS35^+/m^ hippocampal neurons (transfected at DIV 10, and imaged at DIV 15), respectively. Indeed, the spine density labeled by GFP-GluA1 puncta was also less in VPS35-deficient neurons (Fig. 5a, b), providing additional evidence for VPS35-deficiency in neurons to cause an impaired spine maturation. However, this spine deficit was not obvious in VPS35^+/m^ neurons expressing GFP-GluA2 (Fig. 5c). Interestingly, the surface distributions of GFP-GluA1 (labeled by GFP antibody under non-permeable condition), in particularly at the spine sites, were reduced in VPS35-deficient neurons (Fig. 5a, b). We further examined whether endogenous GluA1/A2’s cell surface distribution in VPS35^+/m^ cortical neurons is reduced by cell surface biotinylation assay. The cell surface levels (over total level) of GluA1 and GluA2 were lower in primary neurons from VPS35^+/m^ brain than that of VPS35^+/+^ controls (Fig. 5e, f). These results suggest that VPS35-deficiency in neurons impairs AMPAR surface distribution.

To understand how VPS35 regulates AMPAR trafficking, we asked whether AMPARs (GluA1 and GluA2) interact with VPS35 and are cargos of retromer. Indeed, both GFP-GluA1 and GFP-GluA2 were co-localized with endogenous VPS35 in hippocampal neurons, particularly in the shaft of dendrites (Additional file 2: Figure S2A-S2B). GFP-GluA1 was also detected in the Flag-tagged VPS35 immunoprecipitated complex in HEK293 cells (Additional file 2: Figure S2C). These results support the view for AMPAR (e.g., GluA1) as a cargo of VPS35/retromer.

### Restoration of the spine maturation deficit by expression of GFP-GluA2, but not GFP-GluA1, in VPS35-deficient neurons

**Fig. 6:**
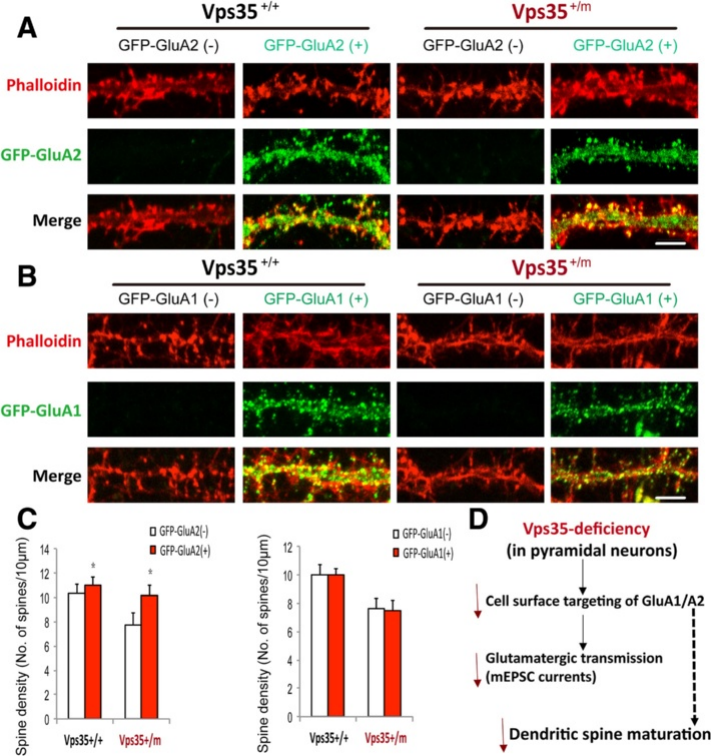
Restoration of the spine deficit of VPS35^+/m^ hippocampal neurons by expression of GluA2, but not GluA1. **a**-**c** Both VPS35^+/+^ and VPS35^+/m^ hippocampal neurons transfected with GFP-GluA1 or GFP-GluA2 at DIV7 were fixed at DIV 15 and stained with phalloidin. **a**, **b**, representative images. Scale bars, 5 μm. **c** Quantification of spine density in A-B. Data were shown as mean ± SEM; *n* = 30 neurons from 3 independent experiments; *p < 0.05. **d** A working model for VPS35-deficiency induced deficits of AMPA receptor trafficking, glutamatergic transmission, and dendritic spine maturation

Note that impairment of AMPARs’ insertion into postsynaptic membrane can affect spine maturation [25–27]. We thus wonder if the impaired GluA1 or A2 targeting to the cell surface may result in the deficit of spine maturation in VPS35-deficient neurons. To address this issue, GFP-GluA1 or GFP-GluA2 was transfected into cultured VPS35^+/+^ and ^+/m^ hippocampal neurons (DIV10). Neurons (at DIV 15) were fixed, stained with phalloidin, and imaged both GFP and phalloidin fluorescence. Remarkably, the spine deficit (e.g., reduced mature spines) due to VPS35-deficiency was restored by expression of GFP-GluA2, but not GFP-GluA1 (Fig. 6a-c). These results suggest that the impaired GluA2 targeting may be responsible for the spine deficit in VPS35-deficient neurons.

## Discussion

The retromer dysfunction is implicated as a risk factor for the pathogenesis of neurodegenerative disorders, including AD and PD [11–16]. The underlying pathological mechanisms appear to be a complex. In this study, we showed that VPS35 regulates GluA1 and GluA2 cellular trafficking (Figs. 5 and 6), and loss of VPS35 function leads to a decrease of cell surface level of GluA1/A2 (Fig. 5), a reduction of glutamatergic neurotransmission (Fig. 3a-c), and an impairment of excitatory synapse/spine maturation (Fig. 3d-h). Expression of GluA2, but not GluA1, partially restore the spine deficit in VPS35-deficient neurons (Fig. 6). Together, our work leads to a working model (depicted in Fig. 6c) that VPS35-deficiency results in an impaired GluA1/A2 cell surface distribution, which may contribute to the reduced neuronal spine maturation and decreased glutamatergic synaptic transmission. This event may also underlie the pathogenesis of neurodegenerative disorders.

Dendritic spines are small actin-rich protrusions that form the postsynaptic part of most excitatory synapses [28]. They are highly dynamic structures and play crucial roles in synaptic functions during learning and memory [28, 29]. We report initially that VPS35 depletion in CA1 neurons by in utero electroporation of miRNA-VPS35 results in a loss of dendritic spines [19]. Here we provide additional evidence for reduced dendritic spines in young adult VPS35-deficient brain and in VPS35-deficient neurons in culture (Figs. 1 and 2). VPS35/retromer regulation of the dendritic spines is of the following characteristics. First, VPS35 is crucial for spine maturation, as the loss of dendritic spines is largely associated with the decrease of mature spines. Second, VPS35 regulation of spine maturation/dynamics appears to be a cell autonomous event, as it is in a brain region and cell type dependent manners and correlates well with VPS35’s expression pattern. This view is also supported by the observation that selectively knocking down of VPS35 expression in CA1 neurons leads to the spine deficit [19].

Although it is unclear exactly how VPS35 regulates dendritic spine maturation/dynamics, the decreased dendritic spines may be due to a defective AMPA receptor protein trafficking and/or actin remodeling in VPS35 deficient pyramidal neurons. Indeed, retromer/VPS35 is implicated in GluA1 and GluA2 receptor trafficking, since it interacts with AMPA receptors [30], and AMPA receptor targeting to the cell surface is impaired (Fig. 5). The retromer/VPS35 is also implicated in actin dynamics, as it interacts with WASH protein complex [31–34], an important complex for actin remodeling, receptor endocytosis, and tubulin cross talk [35, 36]. Thus, it is conceivable that loss of VPS35 function may results in a combination of impairments in both AMPA receptor trafficking and WASH1 mediated actin remodeling in dendrites, leading to a reduction in dendritic spine density and maturation. However, this speculation requires further investigation.

The VPS35^+/m^ brain display an impairment in AMPA receptor-mediated synaptic transmission, which is likely due to a loss of excitatory synapses and a decrease of glutamatergic synaptic activity. Our data corroborate with the view that AMPA receptors, the homo- or heterotetramers of a combination of two of the GluA1-A4 subunits, are the principle ionotropic glutamate-gated receptors mediating excitatory synaptic transmission in the brain [21–24]. How does VPS35/retromer regulate the excitatory synapse numbers and glutamatergic post-synaptic activity? Our studies in cultured neurons showed a reduced cell surface targeting of AMPA receptors (GluA1 and A2) due to VPS35 deficiency (Fig. 5). Expression of GluA2, but not GluA1, in VPS35-deficient neurons partially restored the dendritic spine deficit (Fig. 6). These observations support the view that AMPA receptor trafficking, particularly GluA2, may be critical for dendritic spine maturation.

Our observation of the reduced cell surface targeting of AMPA receptors is in line with the report by Choy et al., that retromer knockdown decreases functional expression of both AMPA and NMDA receptors at synapses [10]. However, it is different from the report that expression of the PD-linked mutation (D620N) in VPS35 results in an increased GluR1 cell surface distribution [30]. These different results suggest that the D620N mutation may behave as a gain of function mutant in this event. However, it remains to be determined if the mutation results in an increased glutamatergic transmission in vivo.

## Conclusion

In aggregate, the results presented here suggest a critical role of VPS35/retromer in regulating AMPA receptor trafficking and dendritic spine maturation, which may be critical for retromer regulation of glutamatergic transmission. Dysregulation of this function may contribute to the impaired glutamatergic transmission associated with the neurodegenerative disorders, including AD and PD.

## Materials and methods

### Reagents and animals

Rabbit polyclonal anti-vps35 antibody was generated by using the antigen of GST–VPS35D1 fusion protein as described [14, 17]. Rabbit polyclonal anti-Synapsin 1a/1b (Santa Cruz), GluR1 (Millipore), GFP (Santa Cruz) antibodies and mouse monoclonal anti-PSD95 (Millipore), GluR1 (Millipore), GluR2 (Millipore), β-actin (Upstate), Flag (Sigma), and TfR (Abcam) antibodies were used.

VPS35 mutant mice were generated by injection of mutant embryonic stem (ES) cells obtained from Bay Genomics as described previously [17, 20]. All experimental procedures were approved by the Animal Subjects Committee at the Georgia Regents University, according to US National Institutes of Health guidelines.

### Expression vectors

The shRNA-VPS35 expression vector was generated by the pSuper vector system, and the miRNA- VPS35 expression vector was generated by the BLOCK-iT Lentiviral miR RNAi expression system (Invitrogen) according to the manufacturer’s instructions [17, 37]. The cDNAs encoding full length VPS35 were amplified by PCR and subcloned into mammalian expression vectors downstream of a signal peptide and a Flag epitope tag (MDYKDDDDKGP) and under control of the cytomegalovirus promoter [38, 39]. The plasmids of GFP-tagged GluA1 and GFP-GluA2 were a gift given by Dr. Richard Huganir (Johns Hopkins University). The plasmid of Synapsin-GFP-actin was a gift given by Dr. Lin Mei (Georgia Regents University). The authenticity of all constructs was verified by DNA sequencing and Western blot analysis.

### Staining

Golgi staining was performed by using the FD Rapid GolgiStain Kit following the manufacturer’s protocal (FD NeuroTechnologies). Spines were counted on secondary and tertiary branches of apical and basal dendrites in the CA1 hippocampal and cortical region individually. 20 segments of either secondary or tertiary dendrites were randomly selected. Dendrites length was measured using ImageJ.

### β-Gal detection

VPS35^+/+^ or VPS35^+/m^ mouse was perfused with 2 % paraformaldehyde and 0.2 % glutaraldehyde in PBS buffer. Brain was removed and post-fixed in the same buffer for 1 h at 4 °C. Sections (30 μm) were incubated in X-gal buffer (2 mM MgCl2, 5 mM potassium ferricyanide, 5 mM potassium ferrocyanide, and 0.1 % X-gal) avoid light at 37 °C for 12 h, washed and mounted in Permount. Images were required by deconvolution digital microscope (Axioplan 2; Carl Zeiss).

### Electrophysiological recording

Hippocampal slices were prepared as described previously (15). After anesthetized with ketamine/xylazine (Sigma, 100/20 mg/kg, respectively, ip), Mice (2 month-old, male) were sacrified, brains were quickly removed into ice-cold modified artificial cerebrospinal fluid (ACSF) containing (in mM): 250 glycerol, 2 KCl, 10 MgSO_4_, 0.2 CaCl_2_, 1.3 NaH_2_PO_4_, 26 NaHCO_3_, and 10 glucose. Coronal hippocampal slices (300 μm) were cut in ice-cold modified ACSF using a VT-1000S vibratome (Leica, Germany) and transferred to a storage chamber containing regular ACSF (in mM) (126 NaCl, 3 KCl, 1 MgSO_4_, 2 CaCl_2_, 1.25 NaH_2_PO_4_, 26 NaHCO_3_, and 10 glucose) at 34 °C for 30 min and at room temperature (25 ± 1 °C) for additional 1 h before recording. All solutions were saturated with 95 % O_2_ /5 % CO_2_ (vol/vol).

Slices were placed in the recording chamber, which was superfused (2 ml/min) with ACSF at 32–34 °C. CA1 pyramidal neurons were visualized with infrared optics using an upright microscope equipped with a 40x water-immersion lens (Axioskop 2 Plus, Zeiss) and infrared-sensitive CCD camera (C2400-75, Hamamatsu). The Pipettes were pulled by a micropipette puller (P-97, Sutter instrument) with a resistance of 3–5 MΩ. Recordings were made with MultiClamp 700B amplifier and 1440A digitizer (Molecular Device). For miniEPSC (mEPSC) recording, pyramidal neurons were holded at −70 mV in the presence of 20 μM BMI and 1 μM TTX, with the pipette solution containing (in mM): 125 Cs-methanesulfonate, 5 CsCl, 10 Hepes, 0.2 EGTA, 1 MgCl_2_, 4 Mg-ATP, 0.3 Na-GTP, 10 phosphocreatine and 5 QX314 (pH 7.40, 285 mOsm). Series resistance was controlled below 20 MΩ and not compensated. Cells would be rejected if membrane potentials were more positive than −60 mV; or if series resistance fluctuated more than 20 % of initial values. All recordings were done at 32–34 °C. Data were filtered at 1 kHz and sampled at 10 kHz.

### Electron micrograph

4 months old mouse were perfused with 4 % paraformaldehyde and 2 % glutaraldehyde in 100 mM sodium phosphate buffer (pH 7.4), then brain was removed and post-fixed in the same buffer overnight at 4 °C. Hippocampus excised from the brain was post-fixed in 2 % osmium tetroxide in NaCac, then stained en bloc with 2 % uranyl acetate, and dehydrated with graded ethanol, embedded with Epon-Araldite resin. Ultrathin sections were prepared by a Leica EM UC6 ultramicrotome (Leica Microsystems) and stained with uranyl acetate and lead citrate. Prepared sections were examined by a JEM 1230 transmission electron microscope (JEOL. USA) at 110 kV with an UltraScan 4000 CCD camera & First Light Digital Camera Controller (Gatan). 30 EM images per mouse were randomly picked and analyzed by investigators unaware of genotypes. Synapses were defined by having alignment of presynaptic and postsynaptic membranes, presynaptic and postsynaptic thickness, and a bunch of synaptic vesicles.

### Immunofluorescence staining and confocal imaging analysis

For immunofluorescence staining of cultured neurons, primary cultured neurons on the coverslips were fixed with 4 % paraformaldehyde at room temperature for 20 min, permeabilized in 0.2 % Triton X-100 for 10 min, after washing with PBS for 3 times, incubated with 5 % BSA in PBS at room temperature for 30 min, and then were incubated with antibodies as previously described at 4 °C overnight. After wash with PBS for 3 times, coverslips were incubated with Alexafluor-conjugated secondary antibodies (1:500, Invitrogen) or Alexafluor-conjugated phalloidin for 1 h at room temperature. Coverslips were mounted with Vectashield mounting medium (Vector). Confocal images were acquired by Zeiss LSM510 confocal microscope by using oil immersion 40 or 63 × objective.

### Cell culture and transfection

HEK 293 cells were maintained in Dulbecco modified Eagle medium (Cellgro) supplemented with 10 % fetal bovine serum (Gembio) and 100U/ml penicillin G and streptomycin (Invitrogen). Transient transfection was performed using polyethylenimine (Aldrich cat. no. 40,872-7) as described [40]. Seventy two h after transfection, cells were subjected to further experiments.

Mouse hippocampal and cortical neurons were cultured from hippocampi or cortices of E18-19 mouse embryos or P0 VPS35^+/+^ or VPS35^+/m^ mice as described [37]. Briefly, tissues were digested in 0.25 % Trypsin at 37 °C for 20 min, then re-suspended in plating medium (DMEM supplemented with 10 % FBS) and plated onto poly-L-lysine coated coverslips in 12 well-plate at a density of 5 × 10^4^ or 1 × 10^5^ per well for 4 h before replacing with maintenance medium (neural basal medium supplemented with B27 and L-glutamax). Maintenance medium was half changed every 3 days. Calcium phosphate method was used to transfect neurons at DIV7-10 as described [37], and at DIV 14–17, neurons were subjected to immunocytochemistry.

### Synaptosomal and PSD fractions preparation

Mouse hippocampi and cortices were homogenized on ice in cold sucrose buffer (0.32 M sucrose and 25 mM HEPES, pH 7.4), and were centrifuged at 500 g for 5 min. The supernatant (S1) were centrifuged at 10,000 g for 10 min to obtain supernatant (S2; light membrane and cytosolic fraction) and the pellet (P2; crude synaptosomal fraction). The P2 fraction was washed twice with cold sucrose buffer and then re-suspended in cold HBS buffer (25 mM HEPES, pH 7.4, and 150 mM NaCl) to get the synaptosomal fraction. For PSD fraction, samples were re-suspended in 1 % Triton HBS buffer at 4 °C for 30 min alternatively, and centrifuged at 10,000 g for 20 min. Both synaptosomal and PSD fractions were dissolved in 2D buffer (30 mM Tris pH 8.5, 5 mM Magnesium Acetate, 8 M Urea, 4 % CHAPS). Proteins were eluted with SDS sample buffer by boiling for 5 min.

### Co-immunoprecipitation

For in vitro co-immunoprecipitation, HEK 293 cells transfected with different constructs were lysed in modified RIPA buffer (50 mM Tris pH7.5, 150 mM NaCl, 0.1 % TritonX-100, 10 % Glycerol, 1 mM NaF, 1 mM PMSF and protein inhibitors) for 10 min at 4 °C, and rotated at 4 °C for 1 h. After centrifugation at 13,000 rpm for 10 min, 500 ul of supernantent from transfected cells were incubated with 1 ul of mouse monoclonal anti-flag antibody (Sigma) and 50 ul Protein G-Agarose (Roche) at 4 °C overnight. Beads were washed three times with Lysis buffer and eluted with SDS sample buffer by boiling for 5 min followed by Western blot.

For in vivo co-immunoprecipitation, mouse hippocampi were homogenized in modified RIPA buffer (50 mM Tris pH7.5, 150 mM NaCl, 0.1 % TritonX-100, 10 % Glycerol, 1 mM NaF, 1 mM PMSF and protein inhibitors) by a glass Teflon homogenizer, and rotated at 4 °C for 1 h. After centrifugation at 13,000 rpm for 10 min. 500 ul of brain lysates were incubated with 2 ul of rabbit polyclonal anti-GluR1 antibody (Sigma) and 50 ul Protein A-Agarose (Roche) at 4 °C overnight. Beads were washed three times and eluted with SDS sample buffer by boiling for 5 min with Lysis buffer followed by Western blot.

### Cell surface biotinylation assay

Transfected HEK 293 cells or primary cultured neurons in 60 mm dish were first washed with ice cold PBS/CM buffer (PBS containing 1 mM MgCl_2_ and 0.1 mM CaCl_2_), and incubated with Sulfo-NHS-LC-Biotin in PBS/CM buffer at 4 °C for 40 min. Washed once with ice cold PBS/CM and incubated with 0.01 M glycine, followed by three washes in ice cold PBS/CM. Cells were harvested in RIPA buffer (50 mM Tris pH7.5, 150 mM NaCl, 1 % TritonX-100, 0.02 % SDS, 1 mM PMSF and protein inhibitors). After centrifugation at 13,000 rpm for 10 min, the supernatant was incubated with 50 ul of 50 % avidin agarose (Thermo) with rotating overnight at 4 °C, followed by three washed with lysis buffer. Bound proteins were eluted with SDS sample buffer by boiling for 5 min. Total protein and isolated biotinylated proteins were analyzed by immunoblotting.

### Statistical analysis

All values were presented as mean ± SEM. Statistical analysis were performed by Student’s *T*-test. The significant level was set when *p* <0.05.

## Additional files

Additional file 1: Figure S1. Decreased VPS35 and reduced mature spines in VPS35^+/m^ neurons. (A) VPS35 signal was reduced in primary hippocampal neurons from VPS35^+/m^ mouse compared with that of VPS35^+/+^ controls. Primary hippocampal neurons were fixed at DIV15 and stained with VPS35 antibody. Scale bars, 5 μm. (B) Spine density was decreased and spine morphology was altered in Synapsin-GFP-actin transfected primary cortical neurons from VPS35^+/m^ mouse, compared with that of VPS35^+/+^ controls. Primary cortical neurons transfected with Synapsin-GFP-Actin at DIV7 were fixed at DIV15. Scale bars, 5 μm. (TIFF 437 kb) Additional file 2: Figure S2. VPS35 interaction with GluA1 and GluA2. (A) Co-immunostaining analysis of endogenous VPS35 with GFP-GluA1/GFP-GluA2 in cultured hippocampal neuron expressed GFP-GluA1/GFP-GluA2. Scale bars, 5 μm. (B) Quantification of colocalization index (overlapping signal over total GFP) in A. Data were shown as mean ± SEM; *n* = 30 neurons from 3 independent experiments; **p* < 0.05. (C) Coimmunoprecipitation analysis of exogenously expressed flag-VPS35 and GFP-GluA1. HEK293 cells transfected with indicated plamids were lysed and subjected to co-immunoprecipitation assays. The resulting lysates were loaded onto SDS-PAGE gels and immunoblotted with indicated antibodies. (TIFF 14153 kb)
