# Supplementary figures and images for: VPS35-deficiency results in an impaired AMPA receptor trafficking and decreased dendritic spine maturation
Source: Mol Brain. 2015 Oct 31;8:70. doi: 10.1186/s13041-015-0156-4 (PMC4628247; doi:10.1186/s13041-015-0156-4)

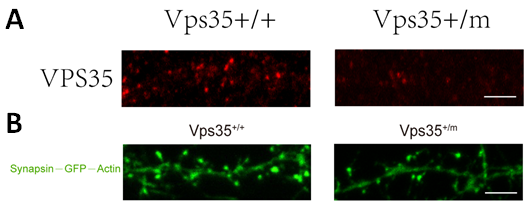

Supplement: Additional file 1: Figure S1. — Decreased VPS35 and reduced mature spines in VPS35+/m neurons. (A) VPS35 signal was reduced in primary hippocampal neurons from VPS35+/m mouse compared with that of VPS35+/+ controls. Primary hippocampal neurons were fixed at DIV15 and stained with VPS35 antibody. Scale bars, 5 μm. (B) Spine density was decreased and spine morphology was altered in Synapsin-GFP-actin transfected primary cortical neurons from VPS35+/m mouse, compared with that of VPS35+/+ controls. Primary cortical neurons transfected with Synapsin-GFP-Actin at DIV7 were fixed at DIV15. Scale bars, 5 μm. (TIFF 437 kb) [file 13041_2015_156_MOESM1_ESM.tif]

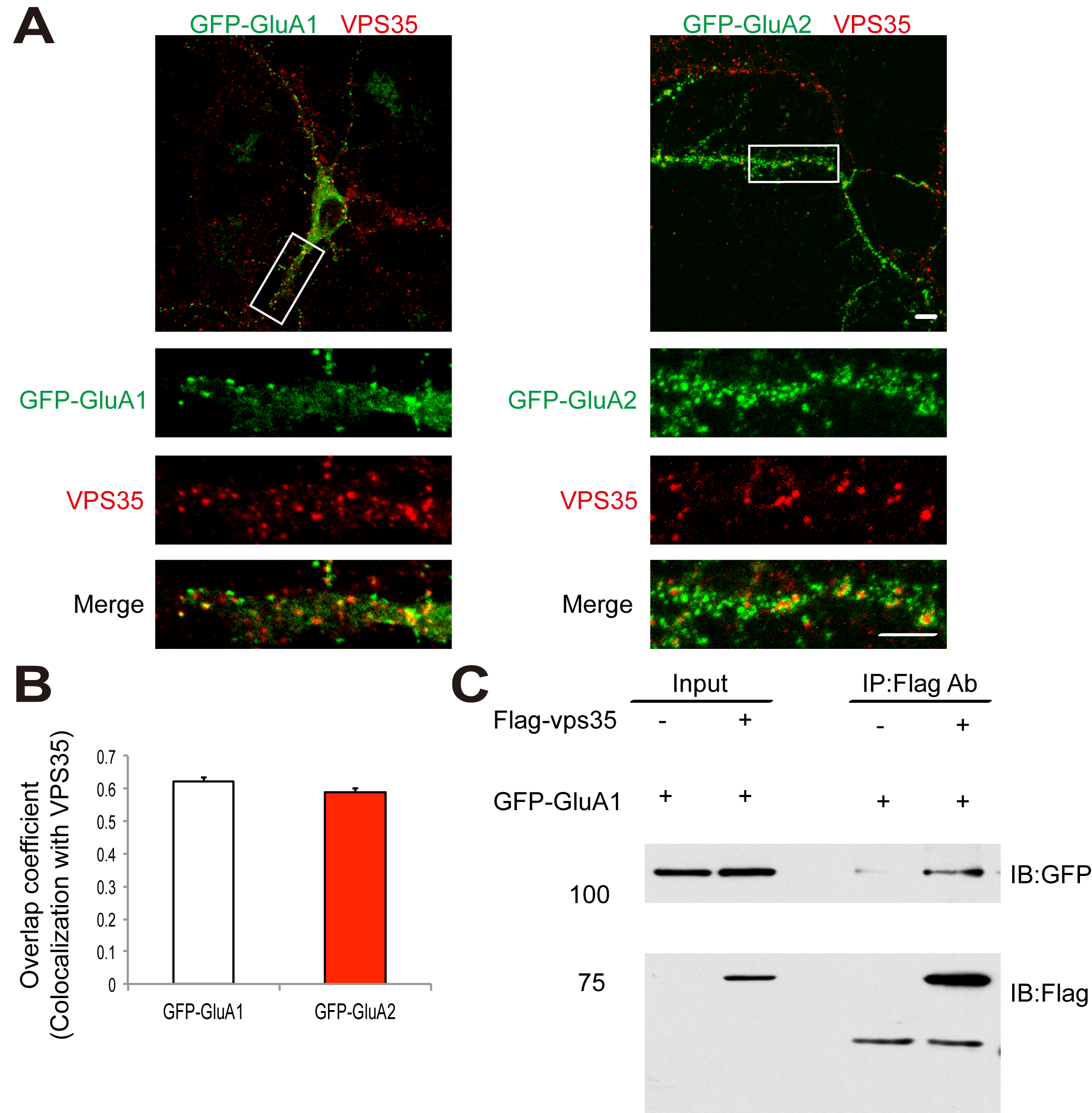

Supplement: Additional file 2: Figure S2. — VPS35 interaction with GluA1 and GluA2. (A) Co-immunostaining analysis of endogenous VPS35 with GFP-GluA1/GFP-GluA2 in cultured hippocampal neuron expressed GFP-GluA1/GFP-GluA2. Scale bars, 5 μm. (B) Quantification of colocalization index (overlapping signal over total GFP) in A. Data were shown as mean ± SEM; n = 30 neurons from 3 independent experiments; *p < 0.05. (C) Coimmunoprecipitation analysis of exogenously expressed flag-VPS35 and GFP-GluA1. HEK293 cells transfected with indicated plamids were lysed and subjected to co-immunoprecipitation assays. The resulting lysates were loaded onto SDS-PAGE gels and immunoblotted with indicated antibodies. (TIFF 14153 kb) [file 13041_2015_156_MOESM2_ESM.tif]
